# Regenerative endodontic therapy option for immature traumatized maxillary permanent incisor: A case report

**DOI:** 10.6026/973206300210675

**Published:** 2025-04-30

**Authors:** Omar AlJasir

**Affiliations:** 1 Department of Conservative Dental Science, College of Dentistry, Qassim University, Kingdom of Saudi Arabia

**Keywords:** Maxillary, permanent incisor, regenerative endodontic therapy, treatment

## Abstract

Regenerative Endodontics (REPs) has been defined as biologically based procedures designed to replace damaged structures. An appropriate treatment for immature, necrotic-pulp includes cleaning and disinfecting the root canal and to promote the growth of pulp-dentin stem cells and to form a blood clot inside the root canal that serves as a natural scaffold.

## Background:

The biologically based techniques known as regenerative endodontics (REP) are intended to restore injured dentin, root structures and pulp-dentin complex cells [1, 2]. It has been proposed as the best course of action for cases involving young, necrotic-pulp permanent teeth. In order to promote pulp-dentin stem cell proliferation and differentiation, it entails cleaning and disinfecting the root canal as well as stimulating the tissue at the root's apex to create a blood clot within the root canal that acts as a natural scaffold [1, 3]. The most frequent reason why root growth in permanent teeth stops is dental trauma. Damage to the anterior teeth may result in pulpal necrosis and tooth displacement, necessitating both orthodontic and endodontic therapy [2, 4]. The advantages of a successful regenerative endodontic treatment (RET) include apical closure (root end development) and/or continuous root development, as well as the eventual avoidance of "traditional" root canal therapy. With a 100% survival rate, this process encourages the growth of roots [5]. Nonetheless, there have been reports of therapy failure with successful retreatment [6, 7]. A case of RET as a retreatment option for an immature, traumatized permanent maxillary incisor is shown in this paper.

## Case report:

A female youngster aged ten reported to primary care facility with illness of fractured maxillary right central incisor (tooth #11). Her maxillary central incisors were impacted by trauma she experienced at the age of eight, according to her prior dental history. In a private clinic, she received dental care. MTA was used to perform a regenerative endodontic retreatment on tooth #11 2 years back but treatment was unsuccessful. When this patient came to our clinic, the radiographic examination revealed an immature open apex, with radiopaque material inside the root canal. The patient's medical history was unremarkable, and the tooth was asymptomatic with a buccal sinus tract (Figure 1(a, b) - see PDF).

A clinical examination showed that the patient responded to the palpation and percussion examinations. Periodontal probing was within normal limit and the tooth exhibited no mobility. The clinical diagnosis was previously treated with chronic apical abscess. Since the tooth was immature, revascularization treatment was planned after informed consent from the parents. After administering local anesthesia, under rubber dam isolation, root canal cleaning and removal of MTA filling was done. With CPR ultrasonic tips, the canal was cleaned with sterile saline solution, irrigated with 20 mL of 5.25% sodium hypochlorite, and flushed with saline to get rid of any remaining root canal filling debris. Calcium hydroxide was applied to the pulp chamber after it had been properly dried with paper points (Figure 1c - see PDF). The sinus tract had healed and the tooth was still asymptomatic two weeks later. After 2 week at second visit 3% mepivacaine hydrochloride was used to anaesthetize the tooth without the use of a vasoconstrictor (Polocaine dental, DENTSPLY Pharmaceutical, York, PA, USA). After setting up a rubber dam, the canal was slowly irrigated with 20 millilitres of 17% EDTA after being cleaned with sterile saline and irrigated with 20 millilitres of 1.5% sodium hypochlorite to eliminate calcium hydroxide. Canal was dried with paper points and apical bleeding was initiated (Figure 2a - see PDF). To serve as a barrier, a layer of "CollaPlug" resorbable matrix (Zimmer Dental, Carlsbad, CA, USA) was applied coronally over the blood clot and placement of Bioceramic putty (Figure 2b - see PDF). A 2 mm layer of glass ionomer was positioned coronal to a 3 mm thick layer of bioceramic putty (Figure 2c - see PDF) followed by composite restoration, Postoperative Periapical radiograph (Figure 2d - see PDF). The patient was asymptomatic at the 3-, 6- and 12-month follow-up (Figure 3a, b, c - see PDF) show healing of the apical radiolucency. The patient reported no recurrence of swelling at the 18 months recall visit, and a periapical radiograph revealed that the root had increase in the length and thickness of the canal walls.

## Discussion:

In immature teeth with pulp necrosis, the goal of RET is to regenerate the pulp-dentin complex so that root development can continue [5]. A 25% of all schoolchildren suffer from dental trauma, according to the International Association of Dental Traumatology (IADT) [8]. In present case, patient had previous dental trauma with noticeable tooth discoloration. Pulp necrosis and pulpcanalobliteration (PCO) are two of the many consequencies that traumatised young teeth may display as a result of dental trauma. Furthermore, a lot of intracanal medications discolour teeth, particularly if they stay in the tooth crown for an extended length of time. The main cause of tooth discolouration is substance leaking in to the dentinal tubules [9, 10]. Calcium hydroxide (CH) was inserted into the canal for a considerable amount of time in our situation. A successful RET is achieved with radiographic proof of periapical healing with increasing root length and root canal wall thickness, representing continuing root formation and the absence of disease (infection) symptoms such as pain, edema or sinus congestion [11]. In the present case, a completeresolutionofintraoral sinus tract was evident. In RET, root canal system disinfection may be more difficult and depends only on intracanal medications and irrigants. At the second appointment, 1.5% NaOCl and 17% EDTA were employed as chemical. It has been proposed that EDTA is the sole irrigants required for pulp regeneration in immature necrotic teeth at the second visit because it increases the continued existence of stem cells of apical papilla (SCAP) with 89% viability [12]. However, in this model, SCAP did not produce any viable cells when irrigation procedures containing 2% CHX were used. Furthermore, compared to EDTA alone, the mixture of NaOCl and EDTA somewhat reduced cell viability [12]. In the current case series, irrigation with 1.5% NaOCl and 17% EDTA had positive effects. Future research should examine the best antibacterial regimen to eliminate the intracanal bacteria. There was a noticeable increase in root length and wall thickness at 18-month recall. Patient did not present any clinical signs or symptoms that would indicate failure of regenerative endodontic retreatment. Similarly, several cases concluded that a second attempt of RET has been a successful approach [13, 14, 15]. The main markers of a successful RET have been identified as the nonexistence of clinical signs and symptoms and radiographic evidence of periapical healing [16]. In the event that an endodontic procedure on an immature permanent tooth fails, this instance demonstrates that RET may be utilized as a retreatment option.
